# Setting Fire to ESA and EMA Resistance: New Targeted Treatment Options in Lower Risk Myelodysplastic Syndromes

**DOI:** 10.3390/ijms20163853

**Published:** 2019-08-07

**Authors:** Anne Sophie Kubasch, Uwe Platzbecker

**Affiliations:** 1 Medical Clinic and Policlinic 1, Hematology and Cellular Therapy, University Hospital Leipzig, 04103 Leipzig, Germany; 2 German MDS Study Group (G-MDS), 04103 Leipzig, Germany; 3 European Myelodysplastic Syndromes Cooperative Group (EMSCO Group), 01067 Dresden, Germany

**Keywords:** MDS, low-risk disease, ESA, EMA, treatment failure

## Abstract

During the last decade, substantial advances have been made in the understanding of the complex molecular, immunological and cellular disturbances involved in the initiation as well as evolution of myelodysplastic syndromes (MDS). In 85% of the mainly frail and older patient population, anemia is present at the time of diagnosis and is thus a major therapeutic challenge. High rates of primary resistance to erythropoiesis-stimulating agents (ESAs), the currently only approved standard therapy to treat anemia in lower-risk MDS, demand the development of novel and efficient drugs with a good safety profile. Luspatercept, a ligand trap of activin receptor II, is able to promote late stage erythropoiesis even in patients failing prior ESA treatment. The presence of ring sideroblastic phenotype defines a subgroup of patients with higher response rates. Additionally, recent developments in clinical research using HIF-1 or telomerase modulation by roxadustat or imetelstat are promising. Other areas of translational research involve targeting the inflammasome by anti-inflammatory drugs in order to improve anemia. These efforts will hopefully pave the way for new targeted treatment options for anemic low-risk MDS patients.

## 1. Introduction

Myelodysplastic Syndromes (MDS) are clonal hematopoietic stem/progenitor cell (HSPC) disorders characterized by ineffective hematopoiesis, peripheral cytopenia, abnormal marrow cell morphology and a risk for transformation to acute myeloid leukemia (AML). With a median age of 71 years at diagnosis, the majorities of individuals with MDS are frail, of older age and often impaired by several comorbidities that may influence outcomes and treatment approaches [1]. Thus, most of these patients are not candidates for intensive therapeutic approaches or allogeneic hematopoietic stem cell transplantation (allo-HSCT), which is a potentially curative therapy for MDS. Diagnosis is currently still based on bone marrow cytology, histology, cytogenetics and flow cytometry, while molecular genetics are increasingly used to predict disease outcome. To find an individual treatment strategy, personalized risk stratification by using the revised International Prognostic Scoring System (IPSS-R) is most important in managing patients and selecting candidates for clinical trials. Current treatment algorithm is risk-adapted, in the two-thirds of low-risk MDS patients (IPSS-R ≤3.5) the main goals of therapeutic strategies are the correction of cytopenia aiming to prevent complications like bleeding or infections, decrease transfusion burden, delay disease progression to higher-risk disease or AML and improve quality of life [1]. In 85% of cases, anemia is present at the time of diagnosis and still a major therapeutic challenge in these patients. Current treatments options include supportive care with regular red blood cell (RBC) transfusions and erythropoiesis-stimulating agents (ESAs) [1]. However, RBC transfusions and chronic anemia are independent risk factors associated with iron overload, resulting in increased cardiovascular risks affecting survival. Thus, treatment of anemia and reduction of transfusion burden are the major therapeutic goals in these lower-risk patient population [1,2].

## 2. Current Standards to Treat Anemia in Low-Risk MDS

### 2.1. Erythropoiesis-Stimulating Agents (ESAs)

In generally, ESAs like recombinant erythropoietin (EPO) and glycosylated forms (darbepoetin) are currently the first-line treatment of anemia in lower-risk MDS. Within around 3 months after therapy initiation, 30–60% of patients experience an erythroid response (Table 1) [3,4], defined by hemoglobin increase of 1.5 g/dL in transfusion-independent [TI] patients or significant reduction or disappearance of transfusion need in transfusion-dependent [TD] patients. In the EPOANE trial, which led to licensing of EPO alfa in the indication of low-risk MDS with anemia, erythroid hematological improvement (HI-E, IWG 2006) was achieved in 32% of EPO treated patients compared to 4% of patients receiving placebo. Transfusion dependence was reduced from 54% to 25% of patients at week 16 to week 24.

Nevertheless, primary resistance to ESA is frequent and most responders relapse in 70% of the cases most likely due to loss of sensitivity of erythroid progenitors to ESAs. Median response duration to ESA treatment is 18 to 24 months [3,4,6,7], in previous studies response was associated with improved survival but had no impact on AML progression [8,9]. The wide range in clinical response rates and duration is attributable to several biological and clinical variables that allow the selection of patients with the highest probability of successful treatment. Better response to ESAs occurs in patients with low baseline endogenous EPO levels (≤200 U/L), low (≤2 RBC units per month) or absent RBC transfusion requirement, normal cytogenetics, marrow blasts <5% and only few (≤2) somatic mutations [8,9,10]. Interestingly, MDS patients with ring sideroblasts (RS) have similar response rates but shorter response duration to ESAs compared to other low-risk MDS [10,11]. According to the “Nordic Score” [8], a predictive tool for response to ESA therapy, patients with pre-treatment low endogenous EPO-level (<500 IU/L) and transfusion burden of less than 4 units within an 8 week period, will respond with a probability of 74% [12]. Beside low endogenous EPO-levels and low transfusion burden, a low IPSS-R was also identified as a predictive factor of response to ESAs. In a previous study analyzing the impact of IPSS-R on response rates, IPSS-R very low, low, intermediate and high-risk groups had 85%, 68%, 48%, and 31% erythroid response rates, respectively [11]. In patients, who either never had or lost response to single agent ESA, the addition of G-CSF may increase response rates in 20–30% of cases [1]. After ESA failure, treatment landscape is disappointing overall, with many patients eventually requiring long-term transfusions due to lack of additional approved therapeutic agents.

#### Mechanisms of Resistance to ESAs

Until now, mechanisms of resistance to ESA are only partially understood. Prior studies showed, that bone marrow cells from MDS patients exhibited an impaired response to EPO in respect of in vitro colony formation. After ESA exposures, burst forming units erythroid (BFU-E) and colony-forming units erythroid (CFU-E) were found to be defective in cultures of unsorted and sorted CD34-positive bone marrow MDS cells [13]. Interestingly, a four-fold higher concentration of recombinant human EPO was required to achieve the half-maximal growth of MDS CFU-E and BFU-E compared to control erythroid progenitors. In MDS, correlation analysis of the relationship between endogenous EPO levels and erythroid progenitors indicated that the development of anemia is not caused by an abnormality in the capacity of EPO to induce the generation of CFU-E. Instead, prior studies showed that a severe deficient BFU-E population is leading to an insufficient influx of EPO-responsive cells after ESA exposure [13]. In line with these results, Frisan et al [14] showed that ESA non-responders had a significantly lower number of BFU-E and CFU-E compared to responders. Thus, the failure of in vitro BFU-E/CFU-E growth in the presence of EPO was a major characteristic of non-responders and level of BFU-E can possibly serve as a prognostic marker for response to ESA treatment in the future [13,14].

**Figure 1:**
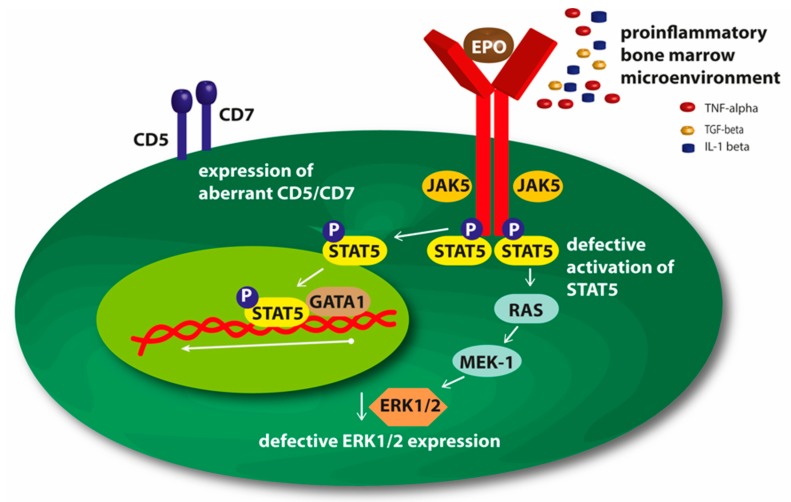
Mechanisms of resistance to erythropoiesis-stimulating agents (ESAs).

Moreover, it has been demonstrated that the percentage of MDS bone marrow cells expressing the EPO receptor (EPO-R) was similar compared to normal marrow cells, also ligand binding capacity of the receptor was intact [15]. Thus it is considered, that a disturbance in an early stage of the EPO signal transduction pathway could be a key factor of resistance to ESA (Figure 1) [14,15,16]. Intracellular structural defects of the EPO-R could cause signal transducer and activator of transcription (STAT) 5-defective activation after EPO stimulation, which leads to an absent or greatly suppressed STAT5 in response to EPO. In earlier studies, reduced STAT5 activation correlated with an impairment of DNA synthesis and with inhibited erythroid colony-forming capacity of MDS marrow cells [16]. Data suggest that not only STAT5 activation is defective in ESA non-responders, they also displayed an impaired phospho (p)-ERK1/2 expression in steady state and after EPO stimulation. By flow cytometry, p-ERK1/2 was significantly lower in bone marrow CD45−/CD71+/GPA−cells from non-responders compared to responders or controls (Figure 1) [17].

Westers et al. investigated the impact of aberrant blast immunophenotypes as a biomarker predicting response to ESA. Immunophenotypic aberrancies (e.g., CD5, CD7, CD56) were mainly found in non-responding patients compared to responders and were therefore highly associated with treatment failure [9]. Oelschlaegel et al. [17] provided some explanation for the above-mentioned theory of phenotypic alterations and subsequent resistance to ESA on the clonal level. The frequency of clonal cells was significantly higher in a subgroup of predominantly lower-risk MDS harboring del(5q) plus an aberrant CD5/CD7 expression compared with IPSS-R matched patients without this aberrant antigen expression [18]. Moreover, they showed that in anemic lower-risk MDS patients with aberrant CD5/7 expression, slightly higher serum EPO levels may cause the significant lower response rates to ESA therapy irrespective of comparable other clinical predictive markers like transfusion burden [18]. Rigolin et al. detected a higher percentage of cytogenetically abnormal karyotypes in patients nonresponsive to ESA [19].

### 2.2. Erythroid Maturation Agents (EMAs)

The erythroid maturation agent (EMA) luspatercept (ACE-536) represents a promising new treatment for patients with lower-risk MDS and RS who require RBC transfusions and are refractory, intolerant or unlikely to respond to ESAs. Luspatercept (ACE-536) and sotatercept (ACE-011) are specific activin receptor fusion proteins, consisting of the extracellular domain of activin receptor IIA (ActRIIA) linked to the human immunoglobulin G1 (IgG1) Fc domain. Activin receptor ligands are members of the TGF-β superfamily which negatively regulate erythropoiesis by induction of apoptosis and cell-cycle arrest in erythroblasts resulting in inhibition of erythroid differentiation. The compounds inhibit the TGF-β pathway by binding to select TGF-β superfamily ligands to reduce aberrant Smad2/3 signaling. After inhibition of Smad-signaling, late stage erythropoiesis such as differentiation of erythroblastst to RBC is promoted [20,21,22].

Luspatercept has already shown promising activity to increase hemoglobin in a phase 2 (PACE-MDS) study in lower-risk MDS patients with transfusion dependent anemia. Patients received luspatercept 1–1.75 mg/kg every 3 weeks subcutaneously for up to 5 cycles [23]. HI-E and RBC-transfusion independence (RBC-TI) responses were 61% (30 of 49 patients) and 55% (16 of 29 patients), respectively. Apart from transfusion burden, the presence of ring sideroblasts (RS), SF3B1 mutation and lower serum EPO levels appeared to define a subgroup with a better response to Luspatercept [23]. These promising findings resulted in the initiation of a placebo-controlled randomized phase 3 study of luspatercept in 229 transfusion dependent low-risk MDS patients with RS or SF3B1 mutation being refractory or not eligible to ESA (MEDALIST trial). 38% and 53% of patients who received luspatercept (1–1.75 mg/kg every 3 weeks) achieved TI and HI-E, respectively, compared to 13% and 12% with placebo (Table 1). During the study, luspatercept had a favorable safety profile and median response duration was 30.6 weeks [24]. Thus, these promising data will hopefully lead to registration of luspatercept by FDA and EMA for this large subset of MDS patients soon. Based on the MEDALIST trial data, a phase III clinical study (NCT03682536, COMMANDS Trial) is currently recruiting and investigates the efficacy and safety of luspatercept versus EPO in ESA-naïve, low-to-intermediate risk MDS patients and transfusion dependence. The study will give answer, whether luspatercept yields higher response rates or longer responses than EPO in this patient population. Because sotatercept (ACE-011) has slightly different affinities than luspatercept to respective ligands, luspatercept rather than sotatercept has been developed in MDS. Further studies are needed to determine mechanism of resistance to luspatercept and to define potential biomarkers of response. Moreover, further clinical trials will answer the question at which treatment stage luspatercept will find his position in the treatment of anemia of non-RS lower-risk MDS.

### 2.3. Immune Modulatory Drugs (ImiDs)

#### Lenalidomide

Until today, ESAs are also first-line option in del(5q) patients with symptomatic anemia and low transfusion burden. Nevertheless, most of these patients initially have excessive EPO levels predicting a short-lived or lack of response to ESAs [1]. For these patients, the immune modulatory drug (ImiD) lenalidomide is still the treatment of choice. Lenalidomide has a high clinical activity in low-risk MDS with del(5q) and a long median response duration of 2 years. Around 50% of patients with de novo MDS have cytogenetic abnormalities, of which del(5q) is the most common. Previous studies demonstrated, that lenalidomide can reduce transfusion requirements and reverse cytologic and cytogenetic abnormalities in patients harboring the 5q31 deletion [25]. The achievement of transfusion independence in 56% to 67% of patients and cytogenetic complete remissions in 45% of patients makes lenalidomide an attractive candidate to treat anemia in transfusion-dependent low-risk MDS with del(5q) (Table 1) [1,26]. Results of the German LEMON5 study showed that patients with del5q and a TP53 mutation displayed overall lower response rates (RBC-TI: 50% vs. 75%) and survival to lenalidomide [27] with a high risk of leukemic progression compared to patients without a TP53 mutation. Lenalidomide has also been investigated in low-risk MDS without del(5q) with response rates of around 25% [28]. However, prognostic biomarkers for response are necessary to define the subset of patients without del(5q) who will respond to lenalidomide treatment. The question, if lenalidomide is able to extend the period of transfusion independency of del(5q) patients will be answered in the recently completed randomized double-blind phase 3 SINTRA-Rev trial (NCT01243476). The study is investigating the efficacy of lenalidomide versus placebo in MDS del(5q) patients without transfusion dependence but hemoglobin value <12 g/dL.

## 3. Novel Targeted Strategies to Treat Anemia in Low-Risk MDS

### 3.1. Roxadustat

Roxadustat (FG-4592) is an orally administered hypoxia inducible factor prolyl hydroxylase inhibitor (HIF-PHIs), currently in clinical development to treat anemia in MDS and chronic kidney disease (CKD) [29]. Activation of hypoxia-inducible transcription factor (HIF) has been identified as an important mechanism of cellular adaptation to low oxygen [30]. Roxadustat promotes erythropoiesis through increasing endogenous EPO levels by stabilization of HIF and improves iron regulation by modulation of hepcidin levels. Phase 1 or 2 data in MDS are not published yet, but administration of roxadustat in mice and rats has shown to improve hemoglobin levels [31]. Currently, roxadustat is under investigation in a phase 3 randomized double-blind placebo-controlled study, analyzing the efficacy and safety of roxadustat to treat anemia in patients with lower-risk MDS and low RBC transfusion burden (NCT03263091) (Table 1).

### 3.2. Imetelstat

Imetelstat is a telomerase inhibitor targeting cells with short telomere lengths and hyperactive telomerase. Administered by intravenous infusion, the compound binds with high affinity to the template region of the RNA component of telomerase, resulting in direct, competitive inhibition of telomerase enzymatic activity [32]. Telomeres are thought to be critical in maintaining normal hematopoiesis and shortening of telomeres leads to reduction of mitotic capacity and consecutive apoptosis. In lower-risk MDS, significantly shorter telomeres compared to healthy controls, higher telomerase activity (TA) and expression of human telomerase reverse transcriptase (hTERT) are linked to a significantly inferior overall survival [33]. A phase 2/3 study investigating imetelstat in RBC transfusion-dependent and ESA-relapsed or refractory lower-risk MDS patients is currently ongoing (NCT02598661). Encouraging preliminary results demonstrated that 37% of patients achieved RBC-TI and 71% of patients achieved HI-E (Table 1) [32,34]. Interestingly, responding patients had reductions in mutational burden and substantial reduction in bone marrow ringed sideroblasts, suggesting potential disease modification potency of imetelstat [32]. These encouraging preliminary results support the planned randomized, double-blind, placebo-controlled phase 3 study to investigate imetelstat in TD low-risk MDS patients relapsed or refractory to ESA treatment, which is expected to start soon.

### 3.3. Immunosuppressive Therapy (IST)

Recently, cell-extrinsic factors promoting inflammation in the bone marrow of MDS patients have been proposed as important mechanisms that contribute to selective competitive advantage of mutant cells to expand and survive [35]. Thus, investigating the synergy between the immune system and the bone marrow mesenchymal stromal cells (MSC) compartment and their impact on early MDS evolution is gaining more and more importance [36]. Especially the interaction between the bone marrow microenvironment and the clonal hematopoietic cells is considered as a central factor of disease evolution [37]. Interestingly, the secretion of TNF-α and other related pro-inflammatory cytokines like IFN-γ, TGF-β, IL-6 are elevated in low-risk MDS, contributing to ineffective hematopoiesis and possibly driving disease progression [5]. Thus, profound immune dysregulation with immune hyperactivation is supposed as a key-feature of early MDS evolution [37]. On the other hand, immunosuppressive cytokines like IL-10 and suppressive Tregs are more intensely secreted in high-risk MDS, the resulting immune subversion may lead to active suppression of a protective immune response and subsequent survival of the malignant clone and disease progression [5]. Therefore, T cell activating therapies like checkpoint inhibitors are currently under investigation in various clinical trials as a possible new treatment approach for high-risk MDS and AML patients. In low-risk MDS the manifestation of cytopenias are in part a result of immune activation and therefore an immunosuppressive therapeutic approach could improve cytopenia and prevent disease progression. Immune modulatory therapies, especially for hypoplastic low-risk MDS, have been used since years. Antithymocyte globulin (ATG, either horse or rabbit), with or without the addition of cyclosporine (CSA) demonstrated trilineage response rates ranging from 16% to 67% [38]. Several patient characteristics have been identified as predictors of response to IST in prior studies, including younger age (<65 years), low blast percentage with hypocellular marrow, limited prior transfusion history (<2 years), good prognostic karyotype and HLA DR15 and PNH clone positivity [39]. Until now, ATG (horse ATG preferred) in combination with CSA is still recommended in clinical routine in rare cases of hypoplastic MDS with normal karyotype. Moreover, the growing evidence that marrow inflammation plays a key role in MDS evolution, opens the window for a broad range of new and potentially powerful targeted treatment options [40]. Prior studies demonstrated that most MDS and AML patients are especially dependent on IL-1 and an IL-1-rich environment, promoting the expansion of malignant progenitors while suppressing normal progenitors [40,41] After cytokine blockade e.g., by monoclonal antibodies, the inhibition of erythroid progenitor colony-forming capacity should be interrupted, resulting in a reactivation of erythropoietin gene expression and improvement of anemia [42]. The development of novel, more personalized and efficient anti-inflammatory drugs is an unmet medical need in this patient population, thus various clinical trials are planned.

## 4. Conclusions

Patients with lower-risk disease, who make up two-thirds of the MDS population, suffer predominately from anemia. Treatment is based on supportive care (i.e., RBC transfusions) and ESAs, the only currently approved treatment option for anemic low-risk MDS patients (1). However, response rates to ESAs are low (below 50%) and mostly only transient. Options for these patients with anemia lacking or losing response to ESAs remain very limited, adding G-CSF to ESA is able to induce responses in 20−30% of cases. Hypomethylating agents (HMA) have also some limited activity in low-risk MDS after failure to first line ESA treatment, but the currently most promising treatment alternative represents luspatercept, a ligand trap of activin receptor II promoting late stage erythropoiesis. Especially in the subgroup of patients with anemia and RS, 40% of patients achieved transfusion independence after luspatercept treatment. Other novel powerful treatment strategies like roxadustat or imetelstat are currently explored in phase 2/3 clinical trials and have so far proved promising. Inflammation of the stem cell compartment and the impact on early MDS evolution is under intensive investigation in pre-clinical and clinical trials and results will hopefully pave the way for new specific targeted treatment options for anemic low-risk MDS patie.

## Figures and Tables

**Table 1:** Targeted treatment options for anemic lower-risk MDS patients.

Therapeutic Compound	Phase	Included Patients	Ongoing/Recent Trial Number	Efficacy	Reference
EPO-α	III	LR-MDS patients with Hb 10 g/dL, serum erythropoietin <500 mU/mL	NCT01381809	HI-E: 45.9% vs. 4.4% (placebo)	[5]
Luspatercept	III	RBC-transfusion depended, RS+ LR-MDS patients	NCT02631070	HI-E: 52.9% vs. 11.8% (placebo)	[5]
Lenalidomide	III	RBC-transfusion depended LR-MDS patients with del5q	NCT00179621	RBC-TI: 42.6–56.1% vs. 5.9% (placebo)	[5]
Roxadustat	III	LR-MDS patients with low RBD-transfusion burden (LTB)	NCT03263091	ongoing	ongoing
Imetelstat	II/III	RBC-transfusion depended LR-MDS patients relapsed/refractory to ESA	NCT02598661	HI-E: 71%, RBC-TI: 37%	[5], ongoing
